# Supplementary material for: Harnessing Skyrmion Hall Effect by Thickness Gradients in Wedge-Shaped Samples of Cubic Helimagnets
Source: Nanomaterials (Basel). 2023 Jul 14;13(14):2073. doi: 10.3390/nano13142073 (PMC10383481; doi:10.3390/nano13142073)
Supplement: Supplementary file 1 [file nanomaterials-13-02073-s001.zip › Supplementary Videos.pdf]

Supplementary Videos

Supplementary Video S1

Simulated current-induced motion of isolated skyrmions and their nucleation in wedge-shaped samples of cubic helimagnets with the elevation angle $\alpha = 10°$. The movie frames are taken with the interval Δt=1ns. Skyrmions nucleated at the sharp boundary also “climb” the hill. The current density, $j_y = 1 \times 10^{13} J/m^2$.

Supplementary Video S2

Skyrmion annihilation at the sharp boundary of the wedge for $\alpha = 25°$. Since the energy minimum of the edge-skyrmion interaction ceases to exist for such large angles, the edge states do not hamper the skyrmion collapse. The current is zero.

Supplementary Video S3

Return of isolated skyrmions into the energy minimum of the edge-skyrmion interaction near the sharp boundary after the current is switched off. $\alpha = 15°$. In this case, the edge states are able to keep the skyrmions within the nanostructure.

Supplementary Video S4

An isolated skyrmion approaches the edge of a racetrack memory with thin-film geometry. The SHE in this case is balanced by the edge-skyrmion repulsion, which leads to the straight skyrmion trajectory. j= $5 \times 10^{12} J/m^2$, the thickness of the layer is 0.5. Notice that according to Fig. 2 in Ref. [54], the skyrmion first moves with variable velocity until it reaches a constant value near the edge.

Supplementary Video S5

For $j = 1 \times 10^{13} J/m^2$, the skyrmion annihilates at the boundary of the racetrack since the repulsive force from the edge state is unable to keep the skyrmion within the sample.
